# Clinical applications of extracellular vesicles: recent advances and emerging trends

**DOI:** 10.3389/fbioe.2025.1671963

**Published:** 2025-10-27

**Authors:** Yitan Zou, Yaping Zhou, Guangyao Li, Yuchao Dong, Shi Hu

**Affiliations:** ^1^ Department of Biomedical Engineering, College of Basic Medical Sciences, Second Military Medical University, Shanghai, China; ^2^ Department of Respiratory and Critical Care Medicine, Shanghai Changhai Hospital, Second Military Medical University, Shanghai, China; ^3^ Department of Gastrointestinal Surgery, The Second Affiliated Hospital of Wenzhou Medical University, Wenzhou, Zhejiang, China

**Keywords:** extracellular vesicles, clinical application, clinical trial, cancer theranostics, MSC-derivedEVs, drug delivery

## Abstract

Extracellular vesicles (EVs) have emerged as pivotal mediators of intercellular communication and promising theranostic agents in medicine. These naturally-derived nanoparticles possess unique advantages including stable physicochemical properties, low immunogenicity, and inherent biocompatibility. In recent years, increasing attention has been directed toward clinical investigations. Driven by these clinical studies, several EV-based diagnostic and therapeutic products have emerged. In this review, we aimed to highlight and discuss the completed and emerging clinical investigations of EV-based strategies, critically elucidate persistent technical and translational hurdles impeding clinical implementation, and propose strategic directions to accelerate realization of the transformative potential inherent in EV-mediated precision medicine.

## 1 Introduction

**FIGURE 1:**
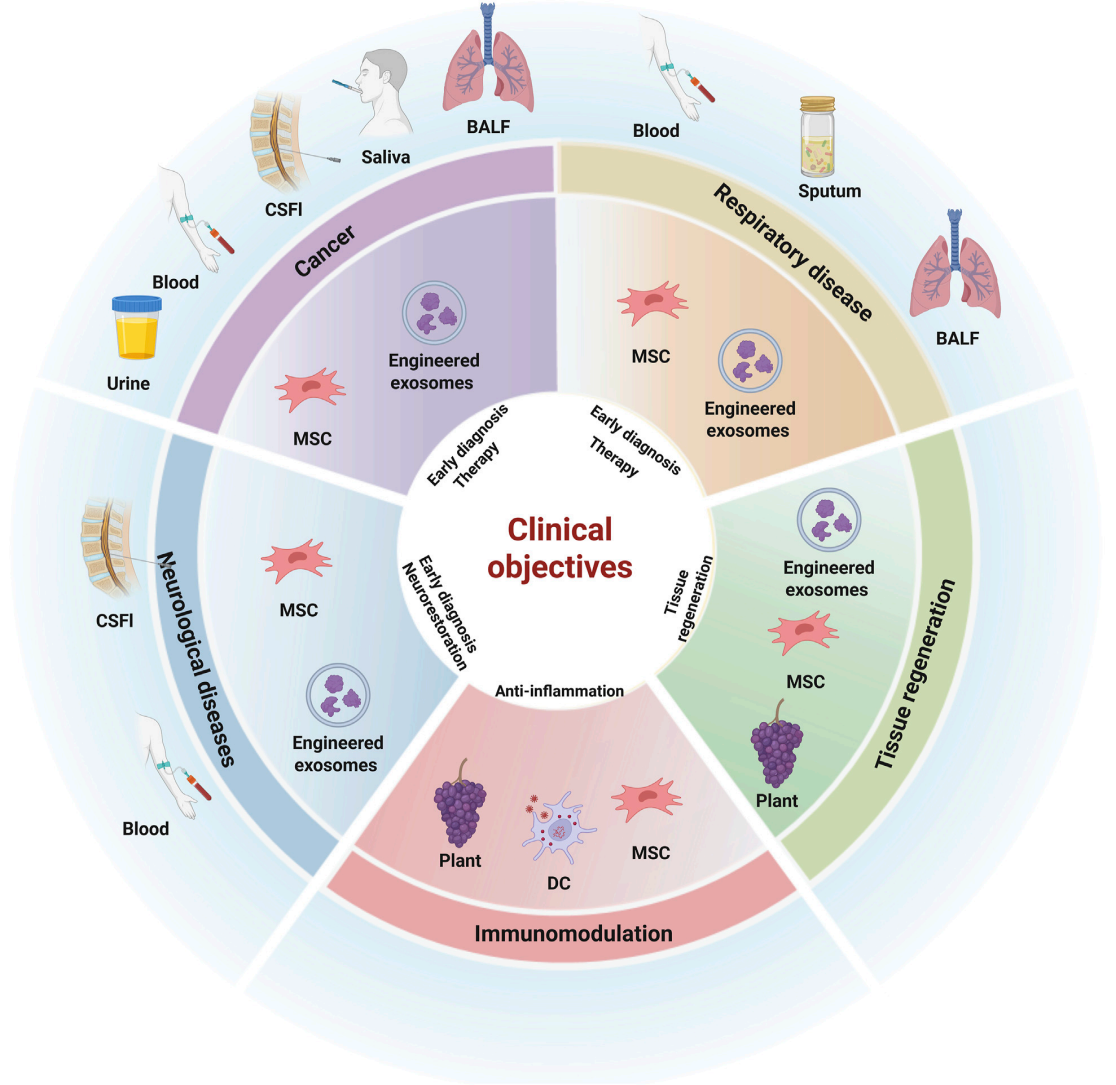
The clinical application of EVs in different kinds of diseases. Created with Biorender.com.

EVs are spherical nanoscale particles with a closed lipid bilayer structure. Based on their formation mechanisms and particle size, they can be classified into exosomes (30–150 nm in diameter), microvesicles (100–1,000 nm in diameter), and apoptotic bodies (1,000–6,000 nm in diameter). EVs are widely present in bodily fluids such as blood, breast milk, saliva, semen, and urine. They play a crucial role in intercellular communication by transporting miRNA, proteins, lipids, and other biological molecules (Wu and Tang, 2025). EVs act as pivotal orchestrators in intercellular communication, playing pivotal roles in numerous physiological and pathological processes like inflammation, tissue repair, immune response and tumor metastasis (Zhang et al., 2024). Therefore, EVs serve diverse and important roles in most biological systems, which has sparked extensive research interest in their applications in fundamental biology, biomarker discovery, and therapeutic applications. Several innate characteristics of EVs implicate their potential diagnostic and therapeutic use. Initially generating from multi-vesicular bodies (MVBs) through inward budding of the intraluminal vesicles, EVs are released from donor cells and serve as a messenger in intercellular communication delivering their cargoes to proximal or distal targeted cells (El Andaloussi et al., 2013). First, EVs show tremendous potential as non-invasive biomarkers for diagnosis especially in malignant tumor (Song et al., 2022). Furthermore, EVs offer a wide range of potential therapeutic uses such as tissue regeneration, immunomodulation and anti-inflammation that exceed traditional cell therapy (Padinharayil et al., 2024). The clinical application of EVs has been boosted by the encouraging results in preclinical studies of different fields, such as cancer, regenerative medicine or immune diseases. Due to the COVID-19 pandemic, the development of clinical-grade EVs and related clinical trials have expanded rapidly over the past 3–4 years, largely promoting EVs research from bench to bedside (Reiner et al., 2017). At the time of writing, more than 200 clinical trials were registered in the US-NIH clinical trial database (https://clinicaltrials.gov, accessed on 7 May 2025) searching for the term extracellular vesicles. Here, we explore the clinical potential of EVs by categorizing diseases and discussing their diagnostic and therapeutic applications within each specific context, based on current and completed clinical trials (Figure 1). Moreover, the challenges and difficulties that hinder the progress of clinical use of EVs are discussed in this review.

## 2 Isolation, characterization, and clinical-grade manufacturing of EVs

Indeed, the production of any investigational medicinal product is governed by Good Manufacturing Practice (GMP) regulations, ensuring that manufacturing and testing comply with established quality standards (Humbert et al., 2025). Achieving the translation of EVs from basic research to clinical application requires standardization and quality control in separation methods, characterization strategies, and clinical-scale mass production.

### 2.1 Isolation methods for EVs

The large-scale downstream isolation of EVs involves diverse methods, with common techniques including: (i) differential ultracentrifugation combined with density gradient centrifugation; (ii) filtration coupled with centrifugation and immunoaffinity capture; (iii) size-exclusion chromatography (SEC); (iv) polymer-based precipitation; and (v) microfluidic technology. It is important to note that each method has distinct strengths and weaknesses depending on the application context, and not all techniques are fully suitable for large-scale purification (Wang et al., 2021; Ferreira et al., 2022; Sun et al., 2024).

### 2.2 Characterization strategies for EVs

Isolated EVs require comprehensive characterization through multiple methods to validate the effectiveness of the isolation technique. Commonly used characterization approaches include: detection of marker proteins, such as transmembrane proteins (CD63, CD9, CD81) and luminal proteins (TSG101, ALIX); analysis of biophysical properties using techniques like dynamic light scattering, flow cytometry, nanoparticle tracking analysis (NTA), and tunable resistive pulse sensing (TRPS); as well as imaging technologies such as atomic force microscopy and electron microscopy. Additionally, the composition of exosomal cargo---including proteins, nucleic acids, lipids, and metabolites---must be characterized via omics methodologies such as proteomics, genomics, and lipidomics to ensure consistency and functionality of their contents (Song et al., 2022).

### 2.3 Clinical-grade production practices for EVs

The clinical-grade production of EVs encompasses both upstream and downstream processes. The upstream process involves the establishment of cell banks, cell expansion, and the preparation of conditioned media. Recent studies indicate that hollow-fiber bioreactors, owing to their unique structure that supports cell attachment and nutrient transport, can be utilized for GMP-compliant exosome production (Ng et al., 2022; Syromiatnikova et al., 2022; Pincela Lins et al., 2023; Kink et al., 2024). Large-scale production must also address challenges related to consistency, efficiency, and cost-effectiveness. Strategies include: optimizing bioreactor culture systems, engineering source cells to enhance proliferation and EV secretion, improving isolation methods, reducing costs, and ensuring regulatory compliance. Additionally, technology transfer plans and personnel training are critical for achieving clinical and commercial production (Jakl et al., 2023).

## 3 Cancer theranostics

Cancer remains a tremendous challenge in medicine due to its complexity, bringing diverse obstacles to diagnosis and treatment (Zhou et al., 2024). Traditionally, disease diagnosis has relied on invasive techniques like tissue biopsies. Traditional cancer diagnosis has often depended on invasive methods such as tissue biopsies. While these techniques provide critical insights, they face limitations, including sampling bias, the requirement for complex surgical interventions, and their inability to monitor disease progression dynamically. These challenges are particularly evident in cancer, where intratumoral molecular heterogeneity further complicates accurate diagnosis (Ilié and Hofman, 2016; Li et al., 2025). Early tumor screening plays a pivotal role in determining optimal therapeutic windows and ultimately shaping clinical outcomes. Contemporary screening protocols predominantly employ radiographic imaging modalities and circulatory tumor biomarker analysis, yet these established techniques exhibit significant limitations in both diagnostic specificity and sensitivity across various malignancies. Blood-based tests, such as liquid biopsies, provide a non-invasive approach for detecting cancer-related biomarkers but are limited by false positives and negatives. Molecular profiling techniques, including next-generation sequencing (NGS) and PCR, enable detailed genetic analysis and support personalized treatment strategies, yet their high cost and technical complexity pose challenges (Vanderpoel et al., 2022). This critical gap in current screening efficacy necessitates the strategic development of next-generation molecular biomarkers with enhanced discriminative capacity. The inherent biomolecular architecture and circulatory dynamics of EVs fundamentally position them as transformative candidates for oncodiagnostic applications, particularly in the emerging paradigm of non-invasive early-detection platforms.

### 3.1 Published clinical trials

To date, only a few EV-based cancer diagnostic tests have received CLIA/FDA approval, among which the ExoDx Prostate IntelliScore (EPI) stands out as one of the rare success stories in translational EV research. The first cancer diagnostic product-ExoDX Lung (ALK) was launched by Exosome Diagnostics in 2016, which is an exosome-based technology detecting RNA and ctDNA simultaneously and performing real-time screening for EML4-ALK mutations in patients with non-small cell lung cancer (NSCLC). ExoDx Lung (ALK) has a sensitivity of 88% and a specificity of 100% in detecting NSCLC and is useful for doctors to determine therapeutic regimen, especially for those patients who are unable or unwilling to accept tissue biopsy (Zhang et al., 2020). The ExoDx Prostate IntelliScore (EPI) Test is a urine exosome gene expression assay to make informed prostate biopsy decisions. A prospective, blinded, randomized, multisite clinical trial was conducted from 2017 to 2018, demonstrating that men receiving EPI low-risk scores (<15.6) significantly defer the time to first biopsy and remain at a very low pathologic risk by 2.5-years after the initial study (Tutrone et al., 2020). Then in 2019, EPI Test was awarded with a Breakthrough Device Designation by the United States Food and Drug Administration (FDA) (Lyu et al., 2023).

### 3.2 Ongoing clinical trials

Up to 7 May 2025, by using the search terms “EVs” or “extracellular vesicles” and “cancer”, we extracted 128 related clinical trials, among which 84 trials focus on EVs application on cancer diagnosis or prognosis and the other 44 used as therapeutic method on cancer. EVs have recently gained attention as promising therapeutic carriers in cancer, leveraging their natural capacity to encapsulate bioactive molecules and deliver them across cellular barriers, including the blood-brain barrier (BBB) (Elsharkasy et al., 2020; Saint-Pol et al., 2020). It has been demonstrated that EVs loaded with therapeutic cargo can enhance treatment response in different cancer (Saari et al., 2015; Zhu et al., 2022; Santos et al., 2023; Tang et al., 2023). Of all the 27 completed clinical trials concerning EVs and cancer, the majority are related to the diagnosis and prognosis of cancer, with only two study focusing on the therapeutic application of EVs in cancer, which suggests that the diagnostic application of extracellular vesicles in tumors is currently more mature, while therapeutic applications are still in the initial stage.

Producing therapeutic EVs presents multiple challenges, particularly in optimizing cargo loading efficiency and achieving precise *in vivo* delivery (Herrmann et al., 2021). EVs can be modified via endogenous or exogenous strategies. Endogenous methods rely on altering parental cells to produce EVs with tailored characteristics, while exogenous approaches involve post-isolation modifications of purified EVs. These strategies are widely investigated for next-generation EV-based drugs, offering improved therapeutic potential.

## 4 Respiratory diseases

EVs can be secreted by various types of cells of respiratory system, including epithelial cells, alveolar cells, immune cells, interstitial cells, inflammation-associated hyperplastic cells and cancer cells in pathological conditions (Li et al., 2021). These EVs can be detected in blood, sputum, or bronchoalveolar lavage fluid (BALF). The acquisition of these samples involves minimally invasive methods, enabling large-scale screening programs and continuous monitoring efforts. Moreover, since the respiratory system directly communicates with the external environment, EVs can be administered not only through traditional bloodstream delivery but also via aerosol inhalation. This approach significantly increases EVs concentration in local lung tissues. Based on the natural characteristics of EVs, an increasing number of studies have emerged to investigate their application in respiratory diseases in recent years such as pulmonary infections, chronic obstructive pulmonary disease (COPD), asthma, acute respiratory distress syndrome (ARDS) and pulmonary fibrosis (PF) (Welsh et al., 2024).

### 4.1 Acute respiratory distress syndrome (ARDS)

Acute respiratory distress syndrome (ARDS) is an acute inflammatory lung injury characterized by diffuse alveolar damage and hyaline-membrane formation with a hospital mortality rate up to 46.1% and no effective pharmacotherapy (Zhuang et al., 2023). The primary clinical management consists of lung-protective ventilation and restrictive fluid strategies (Lewis et al., 2019). MSC-EVs serve as cell-free therapeutic vehicles offering a promising alternative to cellular therapies (Phinney and Pittenger, 2017). The submicron size of EVs renders them ideal candidates for aerosol-mediated delivery in combating respiratory infections (Zubair et al., 2025). Complementarily, MSCs exhibit dual therapeutic actions: suppression of pro-inflammatory cascades and mitigation of oxidative stress/pulmonary fibrogenesis in inflammatory lung diseases (Fujita et al., 2018). The COVID-19 pandemic has greatly promoted the investigation of EV therapy for ARDS over the past 5 years because patients with severe COVID-19 are complicated with cytokine storm and hyperinflammatory syndrome leading to ARDS. A prospective, nonrandomized, open-label, cohort study published in 2020 explored the safety and efficacy of ExoFlo^TM^ in treatment of severe COVID-19 induced ARDS. 24 patients were involved in the trial and were given a single 15 mL intravenous dose of ExoFlo. The results showed that no adverse events were observed and patients’ clinical status and oxygenation improved (Sengupta et al., 2020). After that, a randomized, double-blind, placebo-controlled Phase II clinical trial evaluated the safety and efficacy of ExoFlo™ for treating moderate-to-severe ARDS in COVID-19 patients. The study found that ExoFlo™ (15 mL dose) significantly reduced 60-day mortality in COVID-19-associated ARDS patients aged 18–65 (RR = 0.385, *p* = 0.034) and improved ventilation-free days (*p* = 0.0455), with no treatment-related adverse events observed (Lightner et al., 2023). Another randomized controlled trial published in 2023 evaluated the safety and efficacy of therapy using MSCs and their derived extracellular vesicles in COVID-19-induced ARDS. The result showed that MSCs and their EVs can significantly reduce COVID-19 patients’ inflammatory markers in serum with no serious adverse events (Zarrabi et al., 2023). Due to the disease characteristics of ARDS, clinical research on EVs in this field has focused on improving disease prognosis. In these studies, EVs are primarily derived from mesenchymal stem cells, based on their anti-inflammatory and tissue repair properties, as well as their superior safety and lower immunogenicity compared to whole cells. The differences among these studies lie in the administration methods, observation indicators, dosing frequency, and dosage levels, yet all have yielded positive experimental results. Future efforts will require larger sample sizes and multicenter clinical studies to obtain more reliable evidence-based medical data.

### 4.2 Interstitial lung disease (ILD)

Interstitial lung disease (ILD) represents a large group of progressive pulmonary disorders associated with high morbidity, early mortality and no current approved or golden standard treatment (Johannson et al., 2021). Pulmonary fibrosis represents the final common pathway in the progression of most interstitial lung diseases (Poerio et al., 2023; Heukels et al., 2019). Stem cells possess remarkable capacities for self-renewal and proliferation. Mesenchymal stem cells (MSCs), also referred to as mesenchymal stromal cells, are multipotent cells of mesodermal origin that demonstrate three key biological properties: immunomodulatory activity, anti-inflammatory effects, and most notably, potent antifibrotic capabilities (Yang et al., 2022; Mansouri et al., 2019; Dinh et al., 2020). Cutting-edge research has revealed that EVs serve as pivotal effectors mediating the anti-fibrotic effects of MSCs (Fujita et al., 2018). These EVs can mitigate inflammatory responses and reduce collagen deposition by modulating relevant signaling pathways and cellular polarization, thereby attenuating fibrosis in both airway epithelial cells and pulmonary fibroblasts (Wan et al., 2020). Growing evidence shows that MSC-EVs may have an exclusive role in immune tolerance-autoimmunity balance and preclinical studies have shown the benefits of MSC-EVs therapy for pulmonary fibrosis (Sun et al., 2019). A case report introduced that placenta MSC-EVs treatment for a patient with systemic sclerosis (SSc) complicated by severe ILD resulted in significant improvement in clinical symptoms and reduction in ground glass consolidations and fibrotic changes from chest computed tomography (CT) scans (Assar et al., 2023). Despite promising preclinical evidence in animal models, clinical translation of exosome therapy for fibrotic lung diseases remains limited. To date, only one registered clinical trial has been reported on ClinicalTrials.gov investigating exosome-based therapies for pulmonary fibrosis (NCT05191381). This study initiated in 2021 is currently recruiting participants aged 18–90 years with pulmonary fibrosis after COVID-19 for intravenous administration of MSC-derived EVs. Notably, another registered clinical trial (ChiCTR2300075466) on the Chinese Clinical Trial Registry platform is currently recruiting patients with pulmonary fibrotic lesions confirmed by high-resolution computed tomography (HRCT). This trial investigates the therapeutic potential of nebulized EVs derived from umbilical cord mesenchymal stem cells (UC-MSCs) through inhalation therapy. However, to date, no peer-reviewed publications have emerged from completed clinical studies in this specific therapeutic domain. Preclinical studies and early case reports have demonstrated that MSC-EVs can suppress inflammatory responses, modulate fibrotic pathways, and promote tissue repair (Nataliya et al., 2023). However, no results from completed clinical trials have been published to date, underscoring the need for rigorous clinical validation in this field. Future studies should prioritize large-scale randomized controlled trials to evaluate therapeutic efficacy, optimize delivery methods (e.g., nebulized inhalation vs. systemic administration), and establish standardized protocols for exosome preparation. Despite existing challenges, MSC-EVs represent a promising cell-free therapeutic strategy with significant potential to address unmet clinical needs in progressive pulmonary fibrosis.

### 4.3 Asthma

Asthma is a chronic inflammatory disorder of the airways characterized by variable airflow obstruction and bronchial hyper-responsiveness. Pulmonary cellular networks encompassing both immune effectors (dendritic cells, T/B lymphocytes, regulatory T cells) and structural components (airway epithelium, smooth muscle, fibroblasts) engage in bidirectional exosomal communication. This vesicle-mediated crosstalk dynamically regulates immunophenotypic characteristics and functional responses across mast cells, granulocyte subsets (neutrophils/eosinophils), and tissue-resident cell populations, thereby shaping asthmatic pathophysiology (Fujita et al., 2014). In recent years, increasing evidence have demonstrated the therapeutic potential of MSC-derived EVs in inflammatory lung diseases through inhibition of lung inflammation and vascular remodeling in preclinical studies. Notably, the Chinese Clinical Trial Registry (ChiCTR), one of WHO’s primary registries, recorded its first interventional study (ChiCTR2000031122) on 22 March 2020, investigating MSC-EVs administration in asthma management. This ongoing clinical trial is a randomized parallel-controlled study that will investigate how bone marrow mesenchymal stem cell-derived EVs alleviate airway inflammation and remodeling, and explore the underlying mechanisms. As of now, there have been no relevant literature reporting on clinical trial results regarding exosome-based therapy for asthma. EV-derived therapeutic strategies may offer novel anti-inflammatory approaches for asthma by specifically targeting inflammatory pathways that demonstrate poor responsiveness to glucocorticoid treatment (Park et al., 2025). Numerous preclinical studies have confirmed the therapeutic effects of MSC-EVs in rodent models of asthma. Data indicate that MSC-EVs can reduce the levels of inflammatory cytokines (most notably IL-4) in bronchoalveolar lavage fluid (BALF), improve pulmonary function test results, decrease airway hyperresponsiveness, and reduce the infiltration of inflammatory cells such as eosinophils. Among these, human placental-derived mesenchymal stem cells (H-PMSCs) demonstrated the most significant efficacy (Firouzabadi et al., 2024). A critical limitation in current EV research on asthma involves insufficient systematic evaluation of inter-individual heterogeneity in phenotypic/endotypic characteristics (Wenzel, 2012). The translational gap in preclinical EV research substantially impedes clinical trial development for asthma therapeutics, particularly due to unresolved mechanistic validations and species-specific response disparities.

Building on this, advanced delivery systems need to be developed to enhance lung targeting and prolong drug half-life, thereby maximizing the therapeutic effects of EVs in treating asthma---a chronic inflammatory airway disease.

## 5 Tissue regeneration

Since the 1970s, MSCs have been extensively investigated in regenerative medicine due to their multipotent properties and ability to migrate to injury sites (El Andaloussi et al., 2013). MSCs-EVs demonstrate comparable or enhanced therapeutic effects relative to whole MSCs (Tienda-Vázquez et al., 2023; Zhang and Cheng, 2023). Their advantages include reduced immunogenicity, a safer profile (eliminating risks of uncontrolled differentiation and tumorigenesis linked to cell-based therapies), and greater stability during storage, which alleviates logistical hurdles associated with live-cell preservation (Nooshabadi et al., 2020; Zhang and Cheng, 2023; Tan et al., 2024). In modern medicine, EVs are increasingly used in regenerative therapy, a rapidly advancing discipline focused on repairing and restoring damaged tissues and organs (Sheta et al., 2023). EVs secreted from MSCs showing the potential of promoting angiogenesis represent a cell free therapy in tissue regeneration such as diabetic ulcer, bone regeneration and liver regeneration. Although most application remains in the preclinical stage, clinical trials have begun to conduct preliminary explorations in recent years. A phase I, randomized, double-blind, placebo-controlled trial was published in 2022 evaluating the safety and therapeutic potential of allogeneic platelet-derived extracellular vesicles (pEVs) for delayed wound healing. Although no significant difference in wound closure time was observed between treated and untreated groups due to the rapid intrinsic healing capacity of healthy individuals, the study indicates the potential of pEVs as a scalable therapeutic (Johnson et al., 2023). A case report presents the first successful use of ExoFlo™ to treat a recurrent ischial pressure ulcer in a patients with cerebral palsy. After failing conventional wound care and multiple surgeries, six EV injections over 8 weeks achieved complete healing, with no recurrence for 2 years. This minimally invasive approach offers a promising alternative for patients with limited surgical options due to recurrent ulcers (Messa et al., 2022). One early phase I clinical trial was registered on ClinicalTrial.gov aimed to evaluate the effect of autologous EVs-rich plasma on patients with cutaneous wound healing (NCT02565264). The participants have been treated with specific plasma every day for 28 days and the cutaneous wound healing was evaluated by the length, width and depth of the wound. However, no results have been published from this study. The ClinicalTrials.gov database search demonstrates that to date, there are no registered clinical trials examining the application of MSC-EVs or EVs derived from other sources for the treatment of diabetic cutaneous wounds. In summary, regenerative therapies based on MSCs and EVs demonstrate favorable safety profiles and considerable application potential. However, current research remains largely focused on early-phase trials and case studies, lacking support from large-scale randomized controlled trials. Future efforts should prioritize advancing higher-quality clinical studies to clarify their efficacy, optimize treatment protocols.

## 6 Neurological diseases

Neurodegenerative disorders represent a significant global health challenge, currently impacting millions of individuals across populations. While contemporary therapeutic strategies primarily focus on symptomatic management through palliative interventions, the medical community continues to face critical limitations in developing disease-modifying therapies. The blood-brain barrier (BBB) constitutes a major physiological obstacle in neurological drug development, significantly impeding the transport of pharmacologically active compounds into the central nervous system. Naturally derived EVs, with their inherent biological compatibility, minimal immune reactivity, and unique capacity for BBB penetration, have recently gained prominence as a novel therapeutic delivery platform (Mehdizadeh et al., 2025).

### 6.1 EVs as biomarkers in the diagnosis of neurological disorders

Recent advances have revealed that EVs exhibit source-dependent functional plasticity in neurodegenerative disorders, functioning paradoxically as pathogenic vectors, diagnostic indicators and therapeutic delivery systems. Shi and his colleagues performed a study on the blood of patients with Parkinson’s Disease (PD) to verify the potential of EVs as biomarkers of PD. A total of 267 PD patients and 215 age- and sex-matched healthy controls were enrolled in the trial. They demonstrated that both plasma exosomal α-syn and tau correlated with PD severity, performing even better than the quantification of free α-syn or tau in cerebrospinal fluid (CSF) (Shi et al., 2014). Another clinical study in which 65 patients with acute ischemic stroke (AIS) were enrolled indicated that the expression of serum exosomal miR-9 and miR-124 was increased in patients with ischemic stroke and was also positively associated with infarct volume, serum IL-6 concentration, and National Institutes of Health Stroke Scale (NIHSS) scores. A clinical study assessed the prognostic diagnostic value of circulating EVs in ischemic stroke patients receiving stem cell therapy. In this prospective randomized controlled trial, 39 patients received autologous MSC intravenous injection while another 15 patients as control. It was illustrated that MSC treatment was associated with elevated levels of circulating EVs, which were significantly correlated with improvement in motor function and magnetic resonance imaging (Bang et al., 2022). In summary, as novel diagnostic biomarkers for neurological diseases, EVs demonstrate remarkable sensitivity and specificity in the early diagnosis, disease monitoring, and prognostic evaluation of conditions such as Parkinson’s disease and stroke. They exhibit broad prospects for clinical application and are expanding from diagnostic tools to therapeutic vectors.

### 6.2 EVs as therapeutic agents for neurological diseases

Recent advances indicate that engineered EVs can deliver neuroprotective cargo across the blood-brain barrier, modulate neuroinflammatory pathways, and promote neuroregeneration (Fallahi et al., 2024). A pilot study investigated the efficacy of ExoFlo™ in treating idiopathic and secondary facial paralysis. Seven patients with varying symptom durations and House-Brackmann (HB) grades (III--V) received a 4-week EV protocol involving local perineural injections and intravenous infusions. It demonstrated that EV therapy safely improved facial nerve function in patients with Bell’s palsy and secondary facial paralysis, with all seven participants showing reduced HB scores and enhanced quality of life after a 4-week treatment regimen (Dreschnack and Belshaku, 2023). However, this study did not conduct a statistical comparison with existing treatment options, and therefore lacks support from statistical data. A phase I clinical trial investigated the safety and potential efficacy of intrathecal administration of allogeneic human umbilical cord mesenchymal stem cell-derived EVs (HUC-MSC-EVs) in nine patients with complete subacute spinal cord injury (SCI). The study demonstrated that the treatment was safe, with no adverse events attributed to the intervention. Over a 12-month follow-up, significant improvements were observed in sensory scores, functional independence (SCIM-III total score), and neurogenic bowel dysfunction (NBD score). Motor improvements were noted but not statistically significant. The findings suggest that HUC-MSC-EVs may promote recovery in SCI patients, warranting further phase II/III trials to confirm efficacy. The study highlights the potential of EV therapy as a novel regenerative approach for SCI (Akhlaghpasand et al., 2024). EVs show unique advantages over traditional methods in neurological diseases: they can cross the blood-brain barrier for targeted delivery, regulate neuro inflammation and promote neural repair with high biocompatibility. Their cargos (e.g., miRNAs, proteins) enable precise intervention while minimizing systemic side effects. Current clinical data on extracellular vesicle-mediated drug delivery targeting the central nervous system remain scarce. Most clinical trials on engineered EVs are ongoing, yet published data on their progress or outcomes remain scarce (Li et al., 2023). Translating these therapies into clinics faces major challenges, requiring further research to address scientific and regulatory barriers before their full therapeutic potential can be realized.

## 7 Immunomodulation/anti-inflammation therapies

All of the immune cell types that participate in inflammation can secrete EVs, which in turn have multiple roles in inflammatory processes. EVs have pro-inflammatory roles and anti-inflammatory properties in some environments by the transfer of mediators, danger signals, enzymes and RNAs. Therefore, EVs are considered as attractive candidates for the therapy of autoimmune diseases (Buzas, 2023). Here, we list ongoing or completed clinical trials that aim to harness the theranostics potential of EVs in the field of autoimmune disease (Table 1). This table summarizes 9 registered clinical trials investigating EVs in immune-related diseases, covering classic autoimmune disorders such as Type 1 diabetes mellitus, lupus nephritis, Sjögren’s syndrome, and myasthenia gravis. Among these, 7 observational studies focus on the potential of exosome-carried components (e.g., miRNAs, proteins) for disease prediction and mechanistic exploration, highlighting the clinical translational advantages of EVs as “liquid biopsy” carriers. Only one Phase I trial explores therapeutic intervention using umbilical cord blood-derived mesenchymal stem cell (UCB-MSC) EVs for Type 1 diabetes mellitus. However, its “unknown status” underscores lingering uncertainties in efficacy validation, emphasizing the need for further investigation into the safety and delivery strategies of exosome-based therapies. In the meanwhile, Most trials are in early-phase stages, with nearly half listed as “unknown/not yet recruiting,” suggesting that insufficient sample sizes and the lack of validated biomarker frameworks may hinder clinical translation. Future efforts should prioritize multi-center collaboration and standardized EVs analysis protocols to accelerate the transition from mechanistic research to practical applications.

**TABLE 1:** Observational and clinical trials of extracellular vesicles in autoimmune diseases (from https://clinicaltrials.gov).

Trial identifier	Trial phase	Condition	Trial purpose or intervention	Start date	Status
NCT03984006	Observational	Autoimmune thyroid heart disease	To find earlier predicting biomarkers for heart dysfunction in autoimmune thyroid disease	2019-6-28	Completed
NCT06771427	Observational	Sjogren’s syndrome and dry eye syndrome	To understand the pathological mechanisms of SJS and DES by analyzing the differential proteins in EVs	2025-1-16	Recruiting
NCT05888558	Observational	Ocular muscle myasthenia gravis	To screen serum exosomal miRNA as a biomarker for ocular muscle myasthenia	2023-6-4	Recruiting
NCT04164966	Observational	Type 1 Diabetes	To measure the levels of biomarkers in the body that may indicate the triggers of type 1 diabetes	2019-11-27	Active, not recruiting
NCT06475027	Observational	Sjogren’s syndrome and dry eye syndrome	To detect how acupuncture and Chinese herbal tea bag TBDESJS improve dryness	2024-07-01	Not yet recruiting
NCT02138331	Phase I	Type 1 diabetes mellitus	Treatment with umbilical cord-blood derived MSC EVs	2014-04	Unknown status
NCT04894695	Observational	lupus nephritis	To Identify biomarkers for lupus nephritis by Using Urine EVs	2020-8-2	Unknown status
NCT03106246	Observational	Type 1 diabetes mellitus and type 2 diabetes mellitus	To detect and characterize beta-cell derived EV in blood samples	2016-12	Unknown status

Clinical trials focusing on EVs, in autoimmune diseases were extracted from ClinicalTrials.gov using the search terms “EVs” and “extracellular vesicles”, and filtered for diseases and conditions with autoimmune background.

## 8 Conclusion and future directions

In summary, this review systematically explores the current applications and future prospects of EVs in the field of clinical medicine including EV-based diagnostics and therapeutics, registered clinical trials, and published clinical studies. As naturally occurring intercellular communication vehicles, EVs possess unique characteristics---including excellent biocompatibility, low immunogenicity, the ability to cross biological barriers, and the capacity to carry diverse bioactive molecules (Kooijmans et al., 2016). These properties endow them with immense potential for clinical applications in disease diagnosis, prognosis assessment, therapy, and drug delivery.

First, clinical research on EVs is now being extensively conducted across various disease areas. This trend not only highlights the potential of EVs as novel biomarkers, therapeutic carriers, and regulators in diverse pathological contexts but also reflects their broad applicability and scientific value across diseases. However, significant heterogeneity exists among diseases in terms of pathogenesis, microenvironment regulation, and clinical outcomes, which directly influences the efficacy and safety of exocrine functions and their clinical applications. Therefore, a one-size-fits-all approach cannot be adopted in promoting their clinical translation. Instead, efforts should focus on identifying and selecting specific disease types and patient subgroups most likely to benefit. For example, EVs may serve as ideal drug delivery systems or immunomodulators in certain cancer types, whereas their potential pro-inflammatory or immune-activating risks must be carefully evaluated in autoimmune diseases. Future studies should integrate disease-specific molecular markers, pathological stages, and individual differences to establish evidence-based patient stratification strategies. This will enhance the precision and therapeutic efficacy of exosome applications, ultimately realizing their clinical translational value. Second, standardized quality control frameworks for EVs now have internationally recognized references, most notably the Minimal Information for Studies of Extracellular Vesicles (MISEV) guidelines issued by the International Society for Extracellular Vesicles (ISEV). The MISEV2023 guidelines explicitly point out significant gaps in current exosome research regarding methodology, standardization, and biological understanding, which severely limit the reliability and reproducibility of clinical applications. First, isolation and purification techniques remain inadequate. Current methods such as ultracentrifugation and size-exclusion chromatography struggle to balance high recovery rates with high specificity, often co-isolating contaminants like lipoproteins or protein aggregates that considerably compromise sample purity, especially when using complex biological samples like plasma. Second, characterization systems are seriously insufficient. EVs are highly heterogeneous and lack universal markers. Commonly used protein markers (e.g., CD9, CD63) do not cover all subtypes and cannot effectively distinguish EVs from non-vesicular structures. Significant variations in sensitivity and specificity among detection methods make data comparison and integration challenging. Moreover, sample preprocessing and sources introduce notable variability. Platelet contamination in blood and protein network structures in urine can affect exosome yield and quality. Third, the clinical administration of EVs is a critical factor determining their therapeutic efficacy and safety, requiring systematic and in-depth exploration. The development of an optimal administration strategy must fully consider the pathophysiological characteristics of the target disease, including tissue properties of affected organs, lesion scope, and microenvironment features. Additionally, the pharmacokinetic behavior of EVs---such as organ tropism, retention time, and clearance pathways---should inform rational design of dosing frequency and amount. Current administration methods primarily include systemic delivery and localized targeted delivery. While systemic administration ensures broad distribution, it may lead to rapid clearance by the mononuclear phagocyte system and off-target accumulation in non-target organs, posing potential side effects. Therefore, for central nervous system diseases, localized tissue injuries, or organ-specific tumors, local administration or engineered targeting strategies may be more advantageous. These approaches can enhance drug concentration at the target site while significantly reducing systemic exposure and related risks. Future research should focus on using advanced imaging technologies and biodistribution models to accurately track the *in vivo* fate of EVs under different administration routes. Simultaneously, efforts should be directed toward developing novel engineered drug-loaded EVs and sustained-release delivery systems based on hydrogels, microneedles, and other agents to achieve spatiotemporally controlled drug release. Fourth, advancing EVs from basic research to clinical application depends on in-depth exploration of their mechanisms of action and collaborative efforts in core technology development. Addressing these fundamental scientific questions will provide a theoretical basis for optimizing treatment strategies, predicting efficacy, and mitigating risks, enabling a leap from knowing what they do to understanding how and why they do it. Furthermore, the construction and clinical application of engineered extracellular vesicles (EVs) show great potential for breakthrough improvements in the therapeutic performance of natural EVs. By precisely engineering natural EVs---such as modifying their membrane surfaces with targeted peptide ligands or loading them with specific therapeutic nucleic acids or small-molecule drugs---their accumulation in diseased tissues can be greatly enhanced, their pharmacokinetic properties improved, and off-target effects reduced. This offers a novel strategy for precise and efficient targeted therapy. On another front, artificial intelligence (AI) technology is playing an increasingly important role in the isolation, purification, characterization, and functional decoding of EVs. Traditional analytical methods often fall short when dealing with the high-dimensional, heterogeneous, and complex nature of EV omics data. AI algorithms can efficiently uncover hidden patterns in high-throughput sequencing, proteomics, and single-particle detection data. They hold promise for establishing smarter, more automated EV sorting and identification processes, identifying disease-specific or prognostic EV signature profiles from complex biomarkers, and rationally guiding the design of EV-based therapeutics.

Despite the persistent challenges, research into the clinical application of EVs is advancing at an unprecedented pace. Their revolutionary potential in areas such as non-invasive diagnostics, targeted therapy, and regenerative medicine is now widely acknowledged. With continuous technological breakthroughs, deeper mechanistic understanding, accumulating clinical evidence, and progressively clearer regulatory pathways, we can be confident that EVs will accelerate their transition from laboratory research to clinical practice. This promises new hope and breakthroughs in the diagnosis and treatment of numerous refractory diseases, ultimately benefiting patients worldwide. EVs, as a ‘gift of nature’, face a clinical translation journey that is although not without challenges, offers an immensely promising future, warranting our continued investment and anticipation.
